# Dynamic Chest Radiograph Simulation Technique with Deep Convolutional Neural Networks: A Proof-of-Concept Study

**DOI:** 10.3390/cancers15245768

**Published:** 2023-12-08

**Authors:** Dongrong Yang, Yuhua Huang, Bing Li, Jing Cai, Ge Ren

**Affiliations:** 1 Department of Health Technology and Informatics, The Hong Kong Polytechnic University, Kowloon, Hong Kong; dongrongyang@outlook.com (D.Y.); yu-hua.huang@connect.polyu.hk (Y.H.); zlyylibing4127@zzu.edu.cn (B.L.); 2 Department of Radiation Oncology, Duke University Medical Center, Durham, NC 27708, USA; 3 The Affiliated Cancer Hospital of Zhengzhou University & Henan Cancer Hospital, Zhengzhou 450008, China; 4 The Hong Kong Polytechnic University Shenzhen Research Institute, Shenzhen 518000, China

**Keywords:** chest radiograph, deep learning, motion simulation, lung nodule

## Abstract

**Simple Summary:**

Dynamic chest radiographs offer a distinct advantage over traditional chest radiographs by integrating motion and functional data, elevating their significance in clinical diagnostics. This study introduces a pioneering technique employing deep neural networks to simulate respiratory lung motion and extract local functional details from single-phase chest X-rays, thereby enhancing lung cancer clinical diagnostic capabilities. Our research establishes the viability of generating patient-specific respiratory motion profiles from single-phase chest radiographs. The evaluation of results from the network developed here underscores its substantial accuracy and fidelity, affirming its robustness in providing valuable supplementary insights into pulmonary function.

**Abstract:**

In this study, we present an innovative approach that harnesses deep neural networks to simulate respiratory lung motion and extract local functional information from single-phase chest X-rays, thus providing valuable auxiliary data for early diagnosis of lung cancer. A novel radiograph motion simulation (RMS) network was developed by combining a U-Net and a long short-term memory (LSTM) network for image generation and sequential prediction. By utilizing a spatial transformer network to deform input images, our proposed network ensures accurate image generation. We conducted both qualitative and quantitative assessments to evaluate the effectiveness and accuracy of our proposed network. The simulated respiratory motion closely aligns with pulmonary biomechanics and reveals enhanced details of pulmonary diseases. The proposed network demonstrates precise prediction of respiratory motion in the test cases, achieving remarkable average Dice scores exceeding 0.96 across all phases. The maximum variation in lung length prediction was observed during the end-exhale phase, with average deviation of 4.76 mm (±6.64) for the left lung and 4.77 mm (±7.00) for the right lung. This research validates the feasibility of generating patient-specific respiratory motion profiles from single-phase chest radiographs.

## 1. Introduction

Chest radiography is one of the most frequently used modalities for routine pulmonary disease diagnosis and is commonly utilized as the first exploratory study, as it has the advantages of high availability, low radiation dose, and an efficient workflow. It can suffice as a preliminary examination for conditions including pneumonia, lung cancer, emphysema, and pulmonary fibrosis [1]. In the clinical process, radiologists manually locate anomalies on the radiograph and diagnose the pathology after the picture has been acquired. Then, depending on the circumstances, further diagnostic imaging procedures may be recommended to obtain specialized data for a more precise diagnosis.

Dynamic chest X-ray (DXR) is a functional imaging technique that uses a flat-panel detector (FPD) to generate sequential images. The large field of view and real-time observation can provide information about diaphragm kinetics, pulmonary ventilation, and circulation [2]. It can be deployed as a simple and rapid means of functional imaging, but currently DXR machines have not been widely deployed in clinical practice. Furthermore, the total patient dose of a DXR image is 0.23 mSv, about twice that of a conventional chest X-ray (CXR) [3]. Although dynamic chest X-rays may provide extra functional information, the high dose and scarcity of clinical deployment make it difficult to apply them in routine medical practice. In contrast, conventional chest X-ray machines are widely deployed and expose patients to a lower radiation dose [4]. 

In recent years, advances in engineering and computer science have enabled researchers to reveal a wealth of hidden information encoded in chest radiographs [5], and thereby improve clinical diagnosis. A decrease in X-ray pixel value suggests the presence of a localized air space, which might indicate abnormalities such as a lung cyst, emphysema, bulla, or pneumothorax. Conversely, an increase in the pixel value may indicate a reduction in pulmonary air or intensified tissues, pointing to conditions such as pulmonary inflammation, fibrosis, edema, or sclerosis [6]. By leveraging the capabilities of artificial intelligence (AI), researchers are able to extract a significantly greater amount of information compared to relying solely on observations with the human eye [7]. Approaches like image enhancement [8,9,10,11], organ segmentation [12,13,14], and feature extraction [15,16,17] have been investigated and have achieved promising results. Previous studies have considered whether chest X-rays contain rich three-dimensional and functional information that could be extracted by AI to provide extra information that would improve clinical diagnosis [18]. One previous study tried to predict patients’ lung function to evaluate the need for mechanical ventilation in hospitalized patients based on chest X-ray images and achieved 90.06% accuracy [19]. Liyue et al. explored a deep learning approach for tomographic X-ray imaging with a single-view projection data input, introducing the concept of dimension transformation in the image feature domain to enable volumetric imaging using either single or multiple 2D projections [18]. The feasibility of transforming inter-modality medical imaging with deep learning has also been studied by earlier investigators [20,21].

Motivated by such earlier research, in this study, we utilize the power of deep learning and convolutional neural networks to simulate respiratory lung motion and relative functional information from single-phase digital reconstructed radiographs (DRRs). This technique could be used on conventional chest X-rays to provide auxiliary information for clinical lung cancer diagnosis like dynamic chest X-rays while maintaining the convolutional chest X-ray’s low dosage and high availability.

## 2. Methods

### 2.1. Datasets

In this study, 60 patients’ 4D CT sequences were obtained from several online and local datasets [22,23,24]. The distribution of cases from each dataset is summarized in Table 1. The present study was conducted with the approval of the Institutional Review Board of the Affiliated Cancer Hospital of Zhengzhou University and Henan Cancer Hospital. Due to the scarcity of DXR FPD and DXR data, we generated sequential DRRs from 4D CT to simulate the DXR. During the preprocessing stage, thresholding was first applied to segment the lung volume from the patients’ bodies. With less interference from the contiguous bone components, abnormalities could be readily visualized, allowing the information to be interpreted more precisely. After that, beam eye view (BEV) ray-casting was utilized to generate the DRR. Then, minimum bounding boxes were generated by detecting the boundary of the projected lung volume, and these served as the basis for DRR segmentation. To simulate the screening procedure of chest X-rays, the end-of-inhale (EOI) phase served as the input to the network and the subsequent phases were set as targets. We randomly selected 40 out of 60 patients as the training set, while the remaining 20 patients served as the test set to evaluate the effectiveness of the model.

### 2.2. Network Design

In this study, a novel deep neural network was developed to predict the entire sequence of phases of a complete respiration cycle and the corresponding motion pattern from a single-phase chest X-ray. This technique could be applied to generate auxiliary diagnostic information from a chest X-ray without unnecessary radiation doses or screening time. 

**Figure 1:**
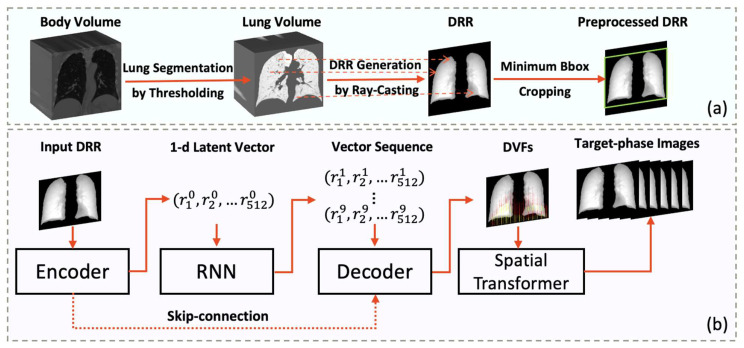
Illustration of the general workflow. (**a**) Preprocessing steps: The lung volumes were initially segmented from the 4D CT body volumes using thresholding. Subsequently, ray-casting was employed to generate the sequential DRR images, which were then cropped to fit the minimum bounding box. (**b**) Sequential prediction steps: The preprocessed DRR images were down-sampled by the encoder to 1D latent vectors. The vectors were then fed into the RNN for sequential prediction. The predicted latent vectors for nine phases were decoded by the decoder to predict the corresponding DVFs. These DVFs were registered to the initial DRR to generate target phase images using a spatial transformer. Abbreviations: DRR—digital reconstructed radiograph; Bbox—bounding box; RNN—recurrent neural network; DVF—deformation vector field.

The general workflow is shown in Figure 1. The development of the proposed radiograph motion simulation (RMS) network mainly comprised two steps: image generation and sequential prediction. To predict the succeeding phases of the respiration cycle $\left(p_{1},p_{2},\ldots p_{9}\right)$ from the EOI phase $p_{0}$, we adopted a recurrent neural network (RNN) embedded U-Net structure [25]. First, the input image $p_{0}\;$ was down-sampled by the encoder block of the U-Net to a one-dimensional feature vector $x_{0}$, which was fed into the RNN for sequential prediction. The decoder block then up-sampled the predicted vector to generate the subsequent phases. Instead of explicitly predicting the images, we used the decoder to generate deformation vector fields (DVFs) and a spatial transformer network to deform the input image to generate accurate medical imaging without sacrificing anatomical detail.

#### 2.2.1. Medical Imaging Generation Model

**Figure 2:**
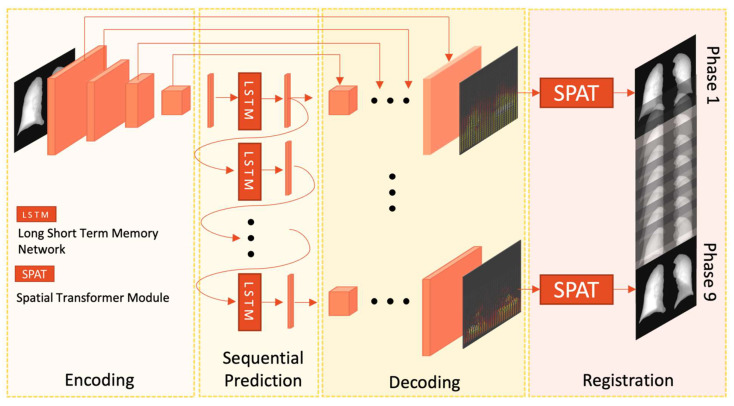
Schematic representation of the advanced RMS framework. The encoding path comprises four successive down-sampling blocks utilizing convolution and max-pooling operations. Sequential prediction is accomplished through a two-layer one-to-many LSTM network. The decoding path is constructed with four symmetrical up-sampling blocks employing de-convolution operations. Additionally, motion registration is achieved simultaneously through a differentiable spatial transformer.

The schematic of the proposed RMS network is shown in Figure 2. The U-Net contracting path consists of four down-sampling blocks. Each block comprises the repeated application of two 3 × 3 convolutions (unpadded convolutions) followed by a rectified linear unit (ReLU) and a 2 × 2 max-pooling operation with stride 2 for down-sampling. Each convolutional operation involves a doubling of the feature channels. After the convolution and max-pooling operation in the contracting path, the down-sampled feature maps are flattened and sent to a fully connected layer to generate a 1 × 512 latent representation. A one-to-many long short-term memory (LSTM) [26] network is then utilized to predict the sequential latent representations. As a kind of recurrent neural network that uses a single input to generate a sequence of outputs, it utilizes the LSTM cells’ ability to maintain an internal state across time steps to selectively retain or discard information. The two-layer network takes as input the previous phase’s latent vector $x_{t-1}$, and outputs the latent vector of the next time step $x_{t}$, which is then fed into the network as the input for the next time step until the maximum time step t=9 is reached. From the LSTM, a sequence of latent representations with 9 phases was generated.

After sequential prediction by the LSTM, the latent vectors were recovered to the same size as the input image through a symmetrically expanding path. Skip-connections between the encoders and decoders ensured information fusion and precise prediction. The expanding path of the U-Net output 9 DVFs ($D_{0},D_{1},D_{2}\ldots D_{9}$) for the subsequent phases of the respiration cycle. A differentiable spatial transformer module [27] was concatenated after the U-Net to deform the initial phase image $p_{0}$ to $p_{1-9}$ according to the predicted DVFs. For each pixel $V_{i}^{t}$ in the target phase image $p_{t}$,

$$V_{i}^{t}=\sum_{n}^{H}\sum_{m}^{W}p_{0}\left(n,m\right)k\left(h_{i}^{t}-m;\Phi_{x}\right)k\left(w_{i}^{t}-n;\Phi_{y}\right)\;\;\forall \;i\in [1\ldots HW]$$

where H and W are the height and width of the initial phase image, and $p_{0}\left(n,m\right)$ is the value at location $\left(n,m\right)$ in the input image. Each $\left(h_{i}^{t},w_{i}^{t}\right)$ coordinate in the corresponding phase’s DVF defines the spatial location in the input image at which a sampling kernel is applied, to obtain the value at a specific output pixel. The $\Phi_{x}$ and $\Phi_{y}$ are parameters of the sampling kernel; we use bilinear interpolation in this study.

After spatial transformation, the discrepancy between predicted phases and the ground truth images was computed and backpropagated to optimize the network parameter.

#### 2.2.2. Loss Function Design

During the optimization, three loss functions were combined to promote precise motion simulation. The first loss function $L_{smooth}$ penalizes the gradient in the predicted DVFs, which aims to foster smoother spatial variations, aligning with the principles of physical feasibility and promoting a more realistic representation of the lung motion.

$$L_{smooth}=\frac{1}{2N}\sum_{t=1}^{9}\sum_{i=0}^{N-1}\left({\left(\frac{\partial D_{t}^{i}}{\partial h}\right)}^{2}+{\left(\frac{\partial D_{t}^{i}}{\partial w}\right)}^{2}\right)$$

This computes the L2 loss of the DVF’s gradient in both directions. N denotes all the pixels in the DVFs and $D_{t}^{i}$ is the ith pixel value in the phase t DVF. 

The second loss function is the mean squared error (*MSE*) loss, measuring the discrepancy between the predicted phase image and the ground truth phase image. It can be expressed as

$$L_{MSE}=\sum_{t=1}^{9}{\left(p_{t}^{gt}-p_{t}^{pre}\right)}^{2}$$

where $p_{t}^{gt}$ is the ground truth image for phase t while $p_{t}^{pre}$ represents the predicted image for phase t. Beyond the *MSE* loss, we also utilized the local cross-correlation loss to improve model robustness, which is computed as

$$L_{NCC}=\frac{{\left(\sum_{i}\left(p_{t,i}^{gt}-{\hat{p}}_{t,i}^{gt}\right)\left(p_{t,i}^{pre}-{\hat{p}}_{t,i}^{pre}\right)\right)}^{2}}{\left({\sum_{i}\left(p_{t,i}^{gt}-{\hat{p}}_{t,i}^{gt}\right)}^{2})(\sum_{i}{\left(p_{t,i}^{pre}-{\hat{p}}_{t,i}^{pre}\right)}^{2}\right)}$$

where $p_{t,i}^{gt}$, $p_{t,i}^{pre}$ denote the ground truth and predicted value of pixel i in phase t, and ${\hat{p}}_{t,i}^{gt}$, ${\hat{p}}_{t,i}^{pre}$ are the corresponding pixel values calculated using the sliding window technique. The final loss was computed as the sum of these three loss functions:

$$L=L_{smooth}+L_{MSE}+L_{NCC}$$

#### 2.2.3. Evaluation Metrics

The accuracy and authenticity of the simulated motion were evaluated using both qualitative and quantitative methods. For qualitative evaluation, the Jacobian determinant of the DVFs was computed. The Jacobian determinant for each pixel shows how much the pixel was stretched or compressed during the deformation, which provides regional information regarding the expansion and contraction of the lung tissue during the respiration cycle to help further identify regions with inadequate or excessive ventilation and assess overall lung function.

$$\;v\left(x,y\right)=J\left(x,y\right)-1=\left[\begin{array}{cc} 1+\frac{\partial u_{x}(x,y)}{\partial x} & \frac{\partial u_{x}(x,y)}{\partial y}\\ \frac{{\partial u}_{y}(x,y)}{\partial x} & 1+\frac{\partial u_{y}(x,y)}{\partial y} \end{array}\right]$$

where *v*(*x*, *y*) is the ventilation of a volume at the point (*x*, *y*); *J*(*x*, *y*) is the Jacobian of the volume at the point (*x*, *y*); and *u*_*x*_, *u*_*y*_ correspond to the x, y components of the DVF, respectively.

For quantitative evaluation, the Dice similarity coefficient (*DSC*) was computed between the predicted images and the ground truth images for each phase.

$$DSC=\frac{2\times |p_{t}^{gt}\cap p_{t}^{pre}|}{\left|p_{t}^{gt}|+|p_{t}^{pre}\right|}$$

Diaphragmatic positions (i.e., the difference between the lung apex and diaphragm dome) were also measured to further analyze the accuracy of the predicted respiratory movements.

#### 2.2.4. Experiment Setup

In this study, we implemented the proposed RMS framework using Python 3.8 and Pytorch 1.12.1 with CUDA 11.6 on a workstation equipped with an NVIDIA GeForce RTX 2080 Ti GPU. The training took approximately two hours. After training, it took less than three seconds to generate the simulated motion per patient.

## 3. Results

**Figure 3:**
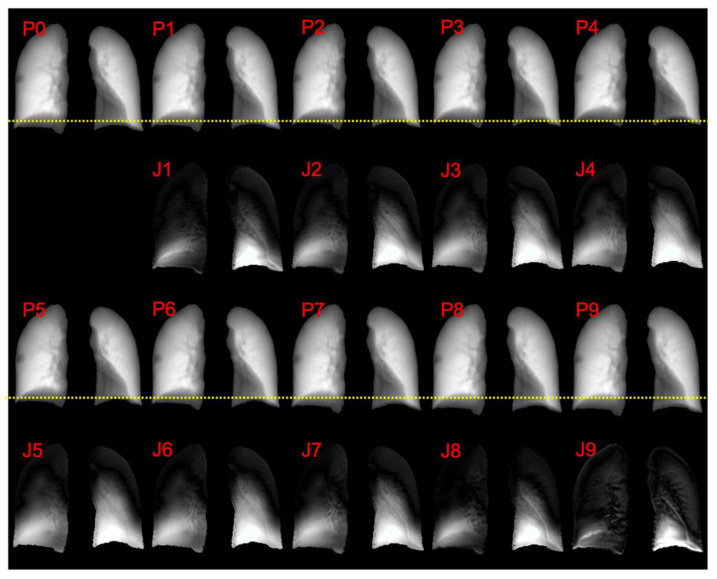
Example test case of respiratory motion simulation. The first and third rows represent the phase images, while the second and final rows display corresponding Jacobian determinant visualizations. Reference lines are shown in the phase images to facilitate a comparison of diaphragm movement between different phases. Abbreviations: P—phase image; J—Jacobian determinant distribution.

For qualitative evaluation, Figure 3 displays the predicted respiratory phases’ images and corresponding Jacobian determinant distribution for one test patient. The movement of the diaphragm can be interpreted by observing the distance between the diaphragm dome and the reference line. During the EOI phase ($p_{0}$) and the end of exhale (EOX) phase ($p_{4}$), as the lung muscles contract, the diaphragm moves upwards, resulting in a significant increase in the distance between the diaphragm dome and the reference. Subsequently, as air is inhaled and the lungs expand, the diaphragm gradually moves downwards, and the distance progressively decreases.

When analyzing the Jacobian determinant distribution, it becomes evident that all phases exhibit a higher contraction factor in the lower lung region compared to the upper lung region. Moreover, phases 4 and 5 demonstrate an overall higher contraction factor compared to the other phases. This observation highlights the regional variations in lung expansion and contraction throughout the respiratory cycle, providing valuable insights into the dynamics of lung function.

**Figure 4:**
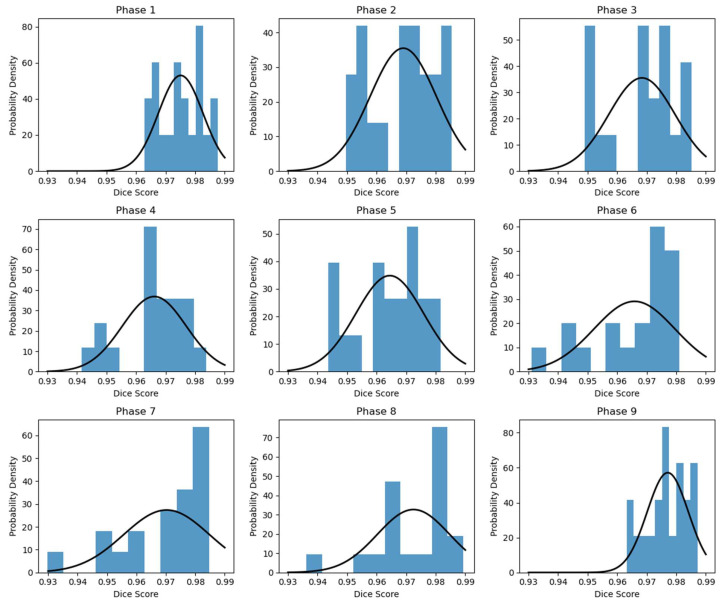
Histogram of phase-based Dice coefficient distribution. For each subfigure, the x-axis represents the Dice score of the predicted image, and the y-axis represents the probability density.

The phase-based Dice coefficient distribution of the test set is shown in Figure 4. For each phase, a normal distribution curve of best fit, with the corresponding mean and standard deviation (std), is included. As shown in the figure, the Dice scores of the phases close to EOI are generally better than those of the EOX phases. Meanwhile, the minimum Dice score of all phases is above 0.93, which indicates that the RMS framework is able to accurately predict the respiratory motion based on the input EOI image.

**Figure 5:**
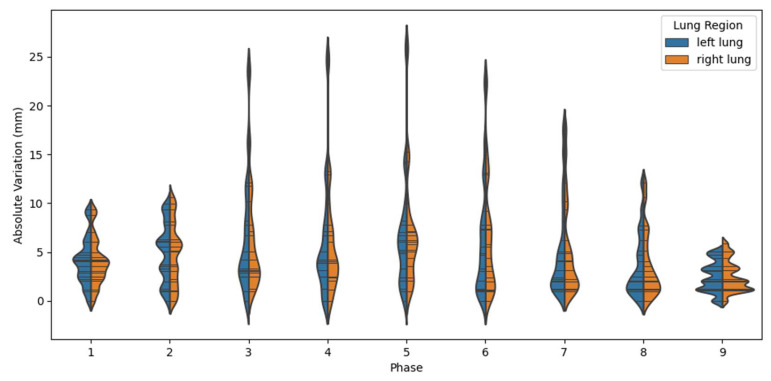
Violin plot of phase-based diaphragmatic position variation. Blue denotes left lung and orange denotes right lung.

The variation in predicted and ground truth diaphragmatic positions is presented in Figure 5. It can be seen that for all phases, the majority of the cases’ variation is less than 10 mm for both lungs. The trend of inter-phase variation is also consistent with the Dice score; the prediction of the end of the sequence is more accurate and precise than the middle of the sequence. 

Table 2 presents the mean and std of the Dice score and diaphragmatic position variation for all phases.

**Figure 6:**
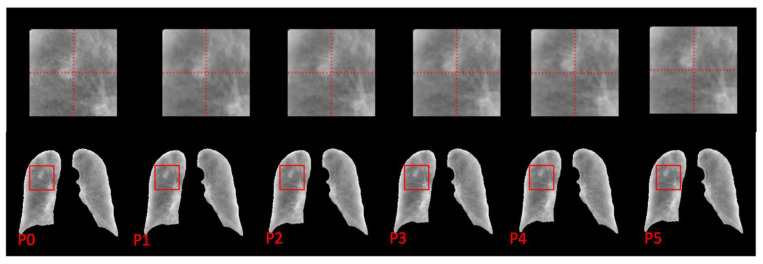
Simulated phase images of a JSRT chest X-ray sample. The region around the nodule was enlarged to improve the visibility of the relative motion.

Given the absence of dynamic chest X-ray data, we conducted our network training using digital reconstructed radiograph (DRR) images to establish proof of concept. Subsequently, we sourced chest X-rays from the JSRT dataset [28] for external validation. These chest X-ray images were processed and integrated into the RMS framework for motion synthesis without further training. A representative example of the motion simulation results from one chest X-ray is illustrated in Figure 6. Notably, the relative motion of a nodule in the right upper lobe becomes readily apparent, alerting radiologists to the presence of abnormal lung function in this specific region.

## 4. Discussions

The chest radiograph continues to be one of the most widely used diagnostic tools for routine pulmonary diseases, for which there has traditionally been a reliance on manual interpretation by radiologists. With the advent of artificial intelligence and computer-assisted diagnosis (CAD) systems, there is persuasive evidence that a wealth of valuable information may be retrieved to improve diagnosis and clinical efficiency [29,30]. While numerous investigations have been made into developing automated classification models based on extracted deep features, the application of AI for providing lung functional information to clinicians remains relatively unexplored. In contrast to directly generating diagnostic decisions, generative models offer additional information that can be harnessed by human radiologists, fostering a more human-centered clinical decision-making process. In this study, we aimed to develop an innovative generative Respiratory Motion Synthesis (RMS) system to generate patient-specific respiratory motion patterns and corresponding deformation vector fields using chest radiographs. The primary objective of this system is to provide clinicians with supplementary functional information, enhancing their understanding of patients’ respiratory dynamics. 

The backbone of the RMS framework is a four-layer U-Net. Initially, the input chest radiographs undergo sequential convolution and max-pooling operations in the encoder blocks, resulting in down-sampled one-dimensional vectors with a length of 512. Subsequently, these vectors are fed into a one-to-many LSTM network, predicting the latent representation of subsequent phases. Then, they are up-sampled by the decoder blocks to generate DVFs with the same size as the input radiographs. Throughout the up-sampling process, skip connections are incorporated to pass features from the encoder path to the decoder path, enabling the recovery of lost spatial information and enhancing model performance. A subsequent spatial transformer network is utilized to deform the input radiograph to the target phases with the predicted DVFs. Compared with directly reconstructing the images from the decoder block, this prevents the loss of anatomical details in the images.

The prediction results of one patient randomly selected from the test set are visualized in Figure 3. It can be quantitively observed that the anatomical details of the lung are well-preserved in the subsequent phases. And the diaphragm motion is shown by the distance between the diaphragm dome and the reference line. The lung tissue’s two-dimensional contraction is visualized by calculating the Jacobian determinant. In the process of exhalation, as respiratory gases are gradually expelled from the lungs, a progressive diminution of the lung volume is observed. This progress of contraction was visualized by sequentially plotting the increasing value of the Jacobian determinant. It can also be observed that the Jacobian determinant of the lower lung region is larger than the upper lung region, representing a higher expansion or contraction factor during respiration, which is consistent with pulmonary biomechanics. The distribution appears smooth without significant fluctuations. The simulation results presented in Figure 6 further substantiate the practicality of integrating this technique into clinical workflows, offering radiologists valuable supplementary information to enhance diagnosis.

For quantitative analysis, the statistics of Dice coefficients between the ground truth and predicted phase images are presented in Figure 4 and Table 2. In the test set, our model consistently attained an average Dice score exceeding 0.96 across all phases, demonstrating its capability to accurately predict overall pulmonary deformation during the respiratory cycle. Notably, when comparing different phases, the inhale phases exhibited generally higher Dice coefficients than the exhale phases. Figure 5 further illustrates a similar pattern in the measurements of diaphragmatic positions. The observed trend could be attributed to increased variation in pulmonary motion and inconsistencies in phase gating. This issue may potentially be mitigated with sequences having a higher imaging frequency and a larger dataset. It also reveals that while most of the test data exhibited errors conforming to a normal distribution with modest mean values, a few data points displayed significant variation across all phases. This discrepancy is likely due to the presence of severe pulmonary diseases, where patients demonstrate relatively extreme motion dynamics that differ from the training dataset, and consequently elude accurate capture by the model. In conclusion, the developed model demonstrates the ability to effectively generate respiratory motion based on single-phase DRR, thereby providing valuable supplementary functional information for diagnosis. This capability holds the potential to enhance clinical efficiency, reduce costs, and facilitate improved patient outcomes.

However, it is important to acknowledge several limitations of this proof-of-concept study. Firstly, the model was trained using digitally reconstructed radiographs (DRRs) generated from 4D CT sequences, rather than sequential chest radiograph datasets. This disparity between the chest radiograph and DRRs may have resulted in a potential decrease in model performance. Secondly, the current dataset size is limited, which could potentially hinder the model’s generalizability across a wide range of clinical conditions. To address these limitations, future efforts will involve acquiring sequential chest radiograph data to fine-tune the model and comprehensively evaluate its performance. By incorporating sequential chest radiographs and expanding the dataset size, we aim to improve the model’s accuracy, robustness, and applicability in real-world clinical scenarios. Another potential area for enhancement is to include a nodule segmentation module in the proposed framework. Currently, in the absence of nodule-specific ground truth annotations within our dataset, our assessment relies on the aggregate motion of the entire lung. Consequently, this approach does not capture the distinct motion characteristics of the nodules. To address this, we intend to collect a dataset with nodule annotations and integrate a dedicated nodule segmentation module to refine the simulation’s precision and improve the accuracy and robustness of the simulation framework.

## 5. Conclusions

This study validated the feasibility of synthesizing patient-specific respiratory motion from a single-phase chest radiograph. Specifically, a novel deep convolutional neural network (RMS) was developed to predict the deformation vector fields of the subsequent phases and deform the initial radiograph with a spatial transformer. The test results of the proposed network have shown promising accuracy and authenticity, confirming its reliability as a tool for extracting additional pulmonary function information. Efficiently providing this information in a human-centered manner, without incurring any additional costs, gives this network the potential to significantly augment the clinical decision-making process. 

## Figures and Tables

**Table 1:** Dataset information.

Dataset Resource	Number of Patients
DIR Lab 4D-CT dataset [22]	10
POPI dataset [24]	5
VAMPIRE challenge 4D CT dataset [23]	12
Henan Cancer Hospital	33

**Table 2:** Phase-based prediction statistics.

Phase #	1	2	3	4	5	6	7	8	9
Dice: mean (±std)	0.975 (±0.0075)	0.969 (±0.0112)	0.968 (±0.0112)	0.966 (±0.0108)	0.964 (±0.0114)	0.966 (±0.0137)	0.970 (±0.0146)	0.972 (±0.0122)	0.977 (±0.0070)
LLE (mm): mean (±std)	4.00 (±2.60)	4.73 (±3.29)	5.80 (±6.42)	4.77 (±6.64)	4.36 (±7.72)	3.09 (±7.98)	1.30 (±7.00)	0.28 (±4.93)	0.36 (±2.89)
RLE (mm): mean (±std)	3.93 (±2.59)	4.62 (±3.47)	5.75 (±6.42)	4.76 (±7.00)	4.29 (±7.92)	3.03 (±7.95)	1.35 (±7.07)	0.18 (±5.00)	0.31 (±2.98)

Abbreviations: LLE—left lung diaphragmatic position variation; RLE—right lung diaphragmatic position variation.

## Data Availability

The datasets generated or analyzed during this study are not publicly available due to restrictions related to patient privacy but are available from the corresponding author on reasonable request.
